# Editorial: Exercise and bone: loading characteristics, mechanisms and adaptation

**DOI:** 10.3389/fphys.2023.1335456

**Published:** 2023-11-16

**Authors:** Zhaojing Chen, Debra Bemben, Katherine Brooke-Wavell

**Affiliations:** ^1^ Department of Kinesiology, California State University, San Bernardino, San Bernardino, CA, United States; ^2^ Department of Health and Exercise Science, University of Oklahoma, Norman, OK, United States; ^3^ School of Sport, Exercise and Health Sciences, Loughborough University, Loughborough, United Kingdom

**Keywords:** exercise, bone, mechanical loading, adaptation, osteoporosis

Osteoporosis is a bone disease characterized by low bone mineral density (BMD) and high risk of fractures, which affects 20% of women and 5% of men aged 50 years and over (Center for Disease Control and Prevention, 2023). Exercise is a key lifestyle intervention for bone health, however, the characteristics of exercise interventions, such as mode, intensity, duration, and volume of exercise, that would be most beneficial to bone health need to be further investigated. To address the gap regarding the bone-loading characteristics, we dedicated a Research Topic on studies specifically investigating effects of loading characteristics, mechanisms of exercise on bone, and subsequent bone adaptation.

Participation of exercise at a young age affects BMD and bone geometry in later life. Using the baseline data from the Bunkyo Health Study, Otsuka et al. found that both men and women who play basketball at young age have higher femoral neck BMD in old age, whereas women who play volleyball at young age have higher lumbar spine BMD in old age. On the other hand, Scorcelletti et al. measured lower limb bone geometry using magnetic resonance imaging and examined the impact of exercise participation on lower limb bone geometry comparing in young and old athletes and sedentary controls. Interestingly, they found that elite track and field athletes have 2° higher femoral frontal bowing regardless of age, but the lower limb geometry was not associated with peak power and force in jumping and hopping.

Sclerostin secreted by osteocytes is a potent antagonist of the Wnt-β-catenin pathway (Delgado-Calle et al., 2017), which is a Research Topic of growing interest in bone research. Mu et al. reported that serum sclerostin concentrations significantly decreased 30-min after ice swimming despite no significant changes in meteorin-like protein, an adipomyokine transforming the white adipose tissue to brown adipose tissue in response to exercise and cold exposure. This conflicts with findings with weightbearing exercise, where a paradoxical increase in sclerostin has been reported (Falk et al., 2016). Mu et al. suggested that the effects on sclerostin could be mediated through endocrine responses to cold and/or exercise, although no correlations were observed in their study. Different from swimming, which is non-weight bearing exercise thought to be less effective on BMD than land-based exercise (Simas et al., 2017), aquatic-based exercise uses devices such as a board or cuff that overcome water resistance in an increasing intensity. Schinzel et al. conducted a meta-analysis and reported that long-term (at least 6 months) aquatic exercise induced significantly greater lumbar spine BMD and femoral neck BMD compared to the control groups.

Blood flow restriction (BFR) is a novel type of training which utilizes a pressure cuff to occlude venous blood flow to the limbs during exercise, and it is often applied with low intensity exercise. Wang et al. focused on the effects of low intensity BFR (walking, plyometric training, and resistance exercise) on bone metabolism including bone turnover markers and BMD. The meta-analysis showed that low intensity resistance training with BFR resulted in greater increases in the bone formation marker (bone specific alkaline phosphatase, BALP), slight increases in BMD, and greater decreases in bone resorption marker (C-terminal telopeptide of type I collagen, CTX) than low intensity resistance training (20%–30% 1-RM), although it is less effective than the high intensity resistance training (60%–80% 1-RM). Walking with BFR had a greater increase in BALP than walking alone.

The mechanisms of bone remodeling are complex and involve many factors. Yi et al. reviewed the role of mitochondrial-derived peptide, MOTS-c, on the regulation of bone metabolism through promotion of osteoblastic proliferation, differentiation and activity and inhibiting osteoclast production. They proposed several potential mechanisms by which exercise may promote bone metabolism through MOTS-c although further experimental evidence is needed.

In conclusion, exercise is a vital intervention to increase bone strength and prevent osteoporosis, however, the bone loading characteristics and effects of novel loading interventions still need further investigation. Articles in this Research Topic have added further to the evidence, reinforcing the importance of exercise during growth on bone shape, providing further evidence for benefits of aquatic and blood flow resisted exercise, as well as adding to literature on possible mechanisms. Original intervention studies in human participants are needed to investigate the optimal exercise program to provide mechanical loading that favorably affects bone metabolism and increases bone strength.
